# Adolescent childbearing trends and sub-national variations in Ethiopia: a pooled analysis of data from six surveys

**DOI:** 10.1186/s12884-018-1917-8

**Published:** 2018-07-03

**Authors:** Yared Mekonnen, Daniel S. Telake, Endeshaw Wolde

**Affiliations:** Mela Research, P.O. Box 34422, Jehovah Nissi Building, Ayat Square, Addis Ababa, Ethiopia

**Keywords:** Ethiopia, Adolescent, Early childbearing, Early marriage, Trends, Determinants

## Abstract

**Background:**

Ethiopia houses the second largest population of female adolescents in Africa. Adolescent childbearing can have detrimental effect to the health and wellbeing of women and their offspring. This study examined trends, sub-national variations and determinants of early childbearing (i.e. childbearing before age 20) in Ethiopia.

**Methods:**

Data from the 2000–2011 Ethiopia Demographic and Health Surveys and from the 2014–2016 Performance Monitoring and Accountability surveys were pooled for this analysis. Based on the year the women reached puberty, five different cohorts were reconstructed that date back to the early 1970s. Kaplan-Meier methodology was used to estimate the cumulative probability of early childbearing and a Cox proportional hazard regression model to examine the associated factors.

**Results:**

The cumulative probability of early childbearing declined by approximately two-fifth in the past four decades, from 57.6 to 35.3%. The occurrence of early childbearing varies substantially by region. In the most recent period, it ranged from 9.6% in Addis Ababa to 59% in Benishangul-Gumuz. Early childbearing risk was reduced by 95% for women who did not marry before the age of 20 years compared to those who married before the age of 18 years. For adolescents who married at the age of 18 and 19 years, early childbearing risk decreased by 60 and 78%, respectively. During the same period, there was a parallel decline in the cumulative probability of early marriage (i.e., before the legal age of 18 years) from 55.3 to 28.7%. Compared with adolescents with no education, those with elementary and secondary or higher education had a 50 and 82% lower risk of early childbearing, respectively.

**Conclusions:**

Early childbearing declined in Ethiopia, largely driven by a parallel reduction in early marriage. However, a large portion of adolescents are still facing early childbearing, and the situation is more dismal in some regions than others. A further reduction in early childbearing is warranted by enforcing the law on the minimum marriage age and expanding secondary and higher education for females. These efforts should give greater emphasis to regions where early childbearing is markedly high.

## Background

Adolescence is the period of transition from childhood to adulthood where major physical, behavioral, social changes occur in the lives of young people. The physical, cognitive, emotional and social transformations that individuals undergo during adolescence form the foundations of their health and well-being later in life [1]. Unfortunately, the picture is bleak for more than half of the world’s adolescents, from ages 10 to 19 years, who live in poor countries [2]. Approximately one in ten babies are born to adolescent mothers globally, with more than 95% of those births occurring in low- and middle-income countries [3].

The consequences of adolescent pregnancy and childbirth are well understood and raise fundamental concerns about the health and social development of young mothers and their children [4]. Indeed, adolescent pregnancy is dangerous, with serious long-term and wide-ranging consequences – from health complications to broader economic concerns [5]. Pregnancy is the leading cause of death for adolescent girls in developing countries, with teen mothers being twice as likely to die from pregnancy-related complications as mothers in their 20s [6]. By impacting education, employment and economic opportunities, pregnancy during adolescence can also have lasting socio-economic consequences, which, in turn, contribute to poorer health outcomes, gender inequity and poverty of adolescent mothers and their families and communities [7, 8].

There are an estimated 6 million adolescent girls in Ethiopia today [9], making the country home to the second largest adolescent population in Africa. As in most sub-Saharan African countries, Ethiopian adolescents are exposed to early marriage, pregnancy and childbirth, which are detrimental to their health and social development and to those of their offspring. The fertility rate of Ethiopian adolescents, aged 15–19 years, is estimated at 79 births per 1000 [10], a rate higher than the world’s average of 46 per 1000 but lower than Africa’s average of 98 per 1000 [11]. Cognizant of this, the government of Ethiopia through its health sector transformation plan [12] and national reproductive health strategy [13] has identified adolescent pregnancy and childbirth as important public health concerns and devised a strategy to address the reproductive health and well-being of Ethiopia’s adolescents.

Understanding the magnitude, trends and determinants of adolescent childbearing can motivate policy and programmatic responses and help monitor progress toward reducing their incidences [14]. There is, however, a general dearth of studies on adolescents’ reproductive and fertility behaviors in Ethiopia, partly because most previous studies have predominantly focused on the fertility behaviors of all women of reproductive age (15–49 years), with little focus on age-disaggregated analysis to address the specific issues that are pertinent to adolescent girls. Furthermore, the few previous studies examining adolescents in Ethiopia have focused on determinants and short-term trends in adolescent childbearing experiences [15–17], as well as the transition to marriage and first sex [18, 19]. Unlike previous studies, our study pooled data from six nationwide surveys that were conducted during the period from 2000 to 2016 and provides a comprehensive and up-to-date analysis of annual trends, determinants and sub-national variations in relation to adolescent childbearing experiences in Ethiopia.

## Methods

### Data sources and inclusion criteria

This study is based on an analysis of pooled national data from six rounds of surveys from two different sources. The data were from the 2000, 2005 and 2011 Ethiopia Demographic and Health Surveys (EDHS) and the 2014, 2015 and 2016 Ethiopia Performance Monitoring and Accountability (EPMA) surveys. These six surveys were selected for this pooled analysis following inventory of all available national and sub-national household surveys that were available in the country at the time this study was initiated and through consultation of the Ethiopia Central Statistical Agency (CSA). The inclusion criteria of the surveys in this pooled analysis were: national and sub-national representativeness of the surveys; similarities in sampling design; the use of the same national sampling frame for clusters selection; samples drawn for the survey by the Ethiopia Central Statistical Agency (CSA); comparability of survey questionnaires for focus variables of this analysis; and availability of raw data for the pooled analysis. Inclusion in the pooled analysis was not restricted by survey date as far as the surveys met the inclusion criteria. Conversely, we excluded any other surveys that did not meet the inclusion criteria.

The Demographic and Health Survey (DHS) is one of the largest programs producing nationally representative household surveys that provide data for a wide range of monitoring and impact evaluation indicators in the areas of population, health, and nutrition throughout the developing world [20]. The Ethiopia DHS is part of the worldwide MEASURE DHS project, which is funded by the United States Agency for International Development (USAID) and other development partners. The country began to implement the DHS in 2000. The survey has been implemented by the CSA, and ICF International provided technical assistance through the MEASURE DHS project. The DHS collected and reported data in five-year intervals.

The EPMA surveys are conducted annually to fill gaps in the timely availability of current and reliable information on population dynamics, reproductive health, family planning, and maternal health, among a few other topics. The lengthy gap between successive DHSs, which restricts the ability of planners and programmers to make timely adjustments to policies and programs based on these data, is the rationale for conducting the PMA surveys [21]. The first EPMA survey was conducted in 2014, which was followed by the 2015 and 2016 rounds. The EPMA is led by the Addis Ababa University’s School of Public Health at the College of Health Sciences, in collaboration with regional universities, the Federal Ministry of Health and the Ethiopia Central Statistics Agency. Overall direction and support is provided by the Bill & Melinda Gates Institute for Population and Reproductive Health at Johns Hopkins Bloomberg School of Public Health and funded by the Bill & Melinda Gates Foundation.

The EDHS and EPMA surveys are based on nationally representative multi-stage cluster sampling, in which the country’s census enumeration areas served as clusters or primary sampling units in both surveys. Both surveys are designed to provide estimates for several indicators for the entire country and, separately, for individual regions. The CSA was responsible for the selection of sample clusters for both surveys using the standard DHS sampling methodology and maintains the comparability of the two surveys. Both surveys raw data with details of data dictionaries and data structures are available in the public domain upon request. Pooling the data from these surveys provides a large data set to allow robust statistical analyses with high precision.

### Data availability

The DHS data used for this study are openly available and can be downloaded at http://www.measuredhs.com/data/available-datasets.cfm?inputSearch=ETHIOPIA. Upon permission from PMA2020, we downloaded the three rounds of the PMA data from http://www.pma2020.org/dataset-download.

### Study variables

Pertinent to this study are the data on the timing of first birth, first marriage, number of children ever born, contraceptive behavior, and basic demographic and socio-economic characteristics of the women, which were gathered from both the EDHSs and EPMA surveys in a similar manner.

The main outcome variable of interest was early childbearing, which was measured by the cumulative probability of having a first birth before the age of 20 years. We also estimated the cumulative probability of having a first birth by a given adolescent age, such as by age 15, 16, 17, 18 or 19 years, based on the responses of women aged 20–49 years who participated in the different surveys.

The data from the six surveys were pooled to create cohorts of women since the early 1970s. For women whose ages ranged from 20 to 49 years in each of the surveys, the year at which they reached puberty (age 10 years) was computed by subtracting the number of years elapsed since they reached an age of 10 years from the date of the surveys. For instance, a woman who was 40 years old in the 2016 survey and another woman who was 35 years old in the 2011 survey both reached puberty in 1986. These women in turn would experience the period of adolescence (age 10–19 years) during the period from 1986 to 1995. Although these women came from two different surveys, they represented a cohort of women who reached puberty around the same year and were thus assigned to the same cohort. Accordingly, five successive cohorts were reconstructed, each representing different periods of entry into the period of adolescence – i.e., 1971–1981; 1982–1987; 1988–1993; 1994–1999; and 2000–2005. The 2000–2005 cohort represents the most recent one and comprised women who were 20–26 years old in 2016. We excluded those women who reached puberty prior to 1971 from the analyses due to the small sample size.

Women who belonged to the same cohort were destined to pass through similar social, economic and related transformations. In this respect, we can mention a few important landmarks that are relevant to the health, population and developmental activities of the country over the past decades and to which the various cohorts were exposed. First, the Ethiopian government issued a number of policies in 1993, including those targeting health, population and women. Second, successive health sector development programs were launched and implemented since the mid-1990s. Third, beginning in the year 2000, the government of Ethiopia has implemented several community-based programs to expand access to primary health care services throughout the country, the most important of which was the launch of the health extension program in 2003.

We examined the relevance of selected background and proximate factors for early childbearing. These factors include the region (categorized into 11 regional states), urban-rural residence, maternal education, and household wealth. We used three categories for women’s educational status: no education, elementary education (1–6 years of schooling), and secondary or higher education (7 plus years of schooling). The EDHS and EPMA survey raw data were provided with wealth index variables that were constructed to rank households using principal component analysis. The wealth quintiles encompassed five categories – poorest, poor, medium, rich, and richest. Proximate factors included in our analyses were marital status and contraceptive use during the adolescent period. We created four marital status categories: (1) married before 18 years old, (2) married at 18 years old, (3) married at 19 years old and (4) not married before 20 years old. This categorization of marital status in part references the Ethiopian minimum marriage age of 18 years. Contraceptive use is a dichotomous variable that measures whether the women used a contraceptive during adolescence (i.e., before 20 years old).

### Method of analyses

The data for the women were transformed into person-years, in which individuals contributed the number of person-years they lived before having a first birth during adolescence. The outcome variable (early childbearing) was dichotomized as 1 or 0. If a woman had her first birth before age 20 years, she was coded as 1; otherwise, she was coded as 0. We used a life-table analysis using Kaplan-Meier (KM) methodology to compute the cumulative probability of having a first birth before 20 years of age. We also estimated the cumulative probabilities of having a first birth at ages of 10, 11, 12…19 years. The median age at first birth was also estimated using the same approach. In addition, the KM method was also used to estimate the cumulative probability of early marriage (i.e., before the legal age of 18 years).

We present temporal trends in the cumulative probability of early childbearing across women’s cohorts at the national level and separately for each region. The trend analysis for each region excluded those women who did not spend their adolescent period (age 10–19 years) in their current place of residence (i.e., current region). The EDHS and EPMA survey collected data for the place of birth, mobility and number of years that the respondent continuously lived in the current place of residence. Using this information, we were able to identify women who spent their adolescent period in the region they resided at the time of the survey and those who did not. Due to the high influx of people to Addis Ababa, the capital city, we did not present a separate trend analysis for the city. The data on mobility revealed that approximately 60% of the women who are currently residing in Addis Ababa were born somewhere else and of whom half did not spend their adolescent years in the city. This result is in stark contrast to the other regions, where approximately 85% of the survey respondents were still living in their birthplaces, and 5% reported that they moved out of their birthplaces but were still living in the same region of other districts. In addition, 6% of the women lived in their birthplaces until 19 years of age before they moved out to other regions of the country. These together comprise 96% of the sample of women who were suitable for the trend analysis. The regional trend analysis excluded women who did not spend their adolescent years in the regions they were residing at the time of the survey.

Temporal trends in the cumulative probability of early childbearing across the cohorts were tested for statistical significance using the log-rank test. To examine determinants of early childbearing, multivariate analyses were performed using the Cox proportional hazards regression model. We present the adjusted hazard ratio (HR) and *p*-value for each covariate. We performed three separate models of background and proximate factors. The first model included only the background factors, i.e., region, education, residence (urban/rural), and wealth. As the main proximate determinant of fertility, we added marital status to the background factors in a second model. The third model additionally included contraceptive use, but it was restricted to only those women who married during adolescence. All three models were applied to the most recent cohort of women.

We used Stata version 12 (Stata Corporation, College Station, TX, USA) for data management and analyses. The Survey command in STATA was used to delineate the strata and primary sampling unit. All proportions, rates and hazard ratios were weighted for the sampling probabilities.

## Results

### Characteristics of the sample

The six surveys collected data from 68,016 women in the reproductive ages of 15–49 years. Excluded from the national level analysis were those women who reached puberty before 1971, resulting in a reduced sample size for this analysis of 50,827 women aged 20–49 years. Furthermore, region-specific trends were examined after excluding those women who did not spend their adolescence in the region they were residing at the time of the interview.

As shown in Table 1, the six surveys contributed to the different cohorts. Naturally, the 2000–2011 EDHSs contributed more data to the earlier cohorts, while the 2014, 2015 and 2016 EPMA surveys contributed more to the recent cohorts. The distribution of the cohorts revealed that 24.2% of the women reached puberty during the years 1971–1981, 20.9% during 1982–1987, 21.9% during 1987–1993, 19.9% during 1994–1999, and 13.1% during 2000–2005. The majority of the sample was from Oromia, followed by the Amhara, SNNP, Tigray and Addis Ababa regions. The other regions combined contributed approximately 7% of the total sample population. This distribution of survey respondents by region compares well across the five cohorts and is a reflection of Ethiopia’s population distribution across the 11 regions [10].

**Table 1:** Number and percent distribution of women who reached puberty (age 10 years) by cohort, according to the source of data (type of survey) and region in Ethiopia

	Cohort: Year woman reached puberty									
	1971–1981		1982–1987		1988–1993		1994–1999		2000–2005	
	N	%	N	%	N	%	N	%	N	%
Survey Year										
2000 DHS	4701	38.2	3598	33.9	1932	17.4	0	0.0	0	0.0
2005 DHS	3589	29.2	2500	23.5	3116	28.0	1386	13.7	0	0.0
2011 DHS	2524	20.5	2328	21.9	2989	26.9	3889	38.4	1454	21.9
2014 PMA	485	3.9	726	6.8	931	8.4	1799	17.7	1341	20.2
2015 PMA	523	4.2	773	7.3	1105	9.9	1575	15.5	2034	30.7
2016 PMA	483	3.9	703	6.6	1057	9.5	1487	14.7	1803	27.2
Total (% from total, *N* = 50,827)	12,304 (24.2)		10,628 (20.9)		11,128 (21.9)		10,135 (19.9)		6632 (13.1)	
Region^a^										
Tigray	1253	6.9	1082	6.5	1091	6.1	1185	6.2	923	6.8
Afar	785	1.3	522	1.0	557	1.0	548	1.1	303	1.1
Amhara	1815	27.9	1450	25.3	1471	24.0	1356	22.7	982	22.0
Oromia	1830	35.4	1591	35.5	1765	37.1	1681	39.0	1221	38.4
Ethiopia-Somali	680	2.1	547	2.2	492	2.0	376	1.7	194	1.2
Benishangul Gumuz	770	1.1	564	1.0	604	1.1	512	1.4	260	1.5
SNNP	1585	20.3	1588	22.7	1699	21.9	1684	20.7	1354	21.9
Gambela	640	0.3	514	0.3	532	0.4	379	0.5	149	0.6
Harari	586	0.3	534	0.3	522	0.3	358	0.3	171	0.4
Addis Ababa	1197	4.0	1179	4.6	1425	5.7	1300	6.0	804	6.0
Dire Dawa	633	0.4	619	0.5	532	0.5	376	0.4	109	0.3

^a^regions’ samples do not add up to the total; those women who did not spend their adolescent period in the current region were excluded

### Trends in early childbearing

**Fig. 1:**
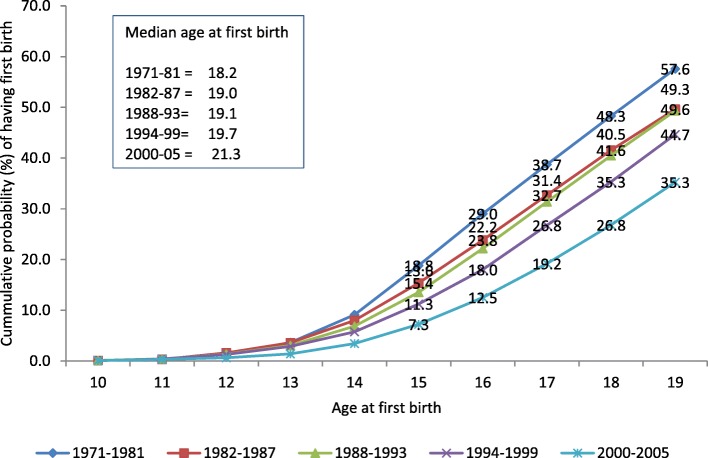
Cumulative probability of having a first birth at different ages and the median age at first birth by cohort in Ethiopia

As shown in Fig. 1, the cumulative probability of having a first birth before the age of 20 years (i.e., before or at age 19 years), defined herein as early childbearing, dropped significantly from 57.6% for the 1971–1981 cohort to 35.3% for the 2000–2005 cohort (*p* < 0.000). This result suggests close to a two-fifths (~ 39%) decline in early childbearing since the early 1970s. The decline was much more apparent for the occurrence of very early childbearing, which occurs in the age range of 15–17 years. For example, the cumulative probability of having a first birth before or at the age of 16 years has declined significantly by more than half from 29 to 12.5% (*p* < 0.000) during the same period. Similarly, the median age at first birth mirrors a significant shift away from early childbirth over the study period; it increased significantly by 3 years over the past four decades – from 18.2 years for the 1971–1981 cohort to 21.3 years for the 2000–2005 cohort.

The trend of the data also revealed that the decline in early childbearing has accelerated since 2000. During the period from 1971 to 1999, early childbearing declined from 57.6 to 44.7%, representing an average annual decline of 0.46%. In contrast, the recorded decline was much faster in the most recent period, especially when data from the last two cohorts were compared – from 44.7 to 35.3%. The average decline between the period from 1994 to 2005 was much faster at 0.85% per annum. Likewise, the median age at first birth during the period from 1971 to 1999 has been staggering, with a slight increase of 1.5 years (from 18.2 to 19.7 years). However, the recent cohort exhibited a relatively faster increase with a median estimated at 21.3 years; since 1994, the median age at first birth has increased by an absolute value of 1.6 years.

Despite the recorded decline, however, early childbearing is still fairly common in Ethiopia, as the most recent cohort (2000–2005) revealed an early childbearing cumulative probability of 35.3%. The corresponding cumulative probability for having a first birth before the age of 18 years was 19.2%.

### Sub-national variations in early childbearing and trends

Table 2 presents the cumulative probabilities of early childbearing by region and according to the cohort. The analyses revealed significant sub-national variations in adolescent childbearing across the cohorts. Sub-national variations in early childbearing have also been increasing over the years. According to the most recent cohort (2000–2005), the cumulative probability of early childbearing ranged from a low of 9.6% in Addis Ababa to a high of 58.9% in Benishangul Gumuz. As suggested by the most recent cohort, three out of the 11 regions had lower values than the national average. The Amhara and Tigray regions demonstrated early childbearing levels approximately equivalent to the national average. Oromia and Harari had a most recent cumulative probability of early childbearing exceeding 40%. Strikingly, four regions suffered from a high level of early childbearing – Ethiopia-Somali (49%), Afar (54.5%), Gambella (57.1%) and Benishangul Gumuz (58.9%).

**Table 2:** Trends in the cumulative probability (%) of early childbearing by cohort and region in Ethiopia

–	Cohort: Year women entered into puberty (age 10 years)					Relative Change (%)^a^	*P*-value (log-rank test)
	1971–1981	1982–1987	1988–1993	1994–1999	2000–2005		
Ethiopia	57.6	49.6	49.3	44.7	35.3	−38.8	0.000
Region							
Dire Dawa	46.3	31.8	32.1	36.7	26.6	−42.5	0.050
SNNP	53.7	48.1	48.6	42.2	31.9	−40.6	0.000
Amhara	67.6	65.2	64.7	55.3	35.9	−47.0	0.000
Tigray	61.9	59.1	58.3	46.6	35.9	−42.0	0.000
Harari	56.8	41.8	36.8	42.5	40.4	−29.0	0.131
Oromia	62.2	52.9	54.3	50.8	40.4	−35.1	0.000
Ethiopia-Somali	46.3	46.3	47.6	51.3	49.0	+ 5.7	0.266
Afar	52.5	51.5	53.5	50.0	54.5	+ 3.8	0.000
Gambela	58.9	56.0	61.5	58.8	57.1	−3.2	0.067
Benishangul Gumuz	65.1	62.2	63.1	61.7	58.9	−9.5	0.025
Addis Ababa^b^					9.6		

^a^Relative change = [(cumulative probability: 2000–2005 – cumulative probability: 1971–1981) / cumulative probability: 1971–1981];

^b^Past trends cannot be examined in Addis Ababa as described in the Methods section

The trend analyses by region excluded Addis Ababa for the reason described above in the Methods section. Six of the 10 regions have seen significant declining trends in early childbearing over the years, although the rate of decline has varied by region. The highest decline occurred in the Amhara region from 67.6 to 35.9%, followed by Dire Dawa from 46.3 to 26.6%, Tigray from 61.9 to 35.9% and SNNP from 53.7 to 31.9%. Oromia also registered a significant decline from 62.2 to 40.4% during the period. Stable or somehow reversed trends in early childbearing can be noted in Ethiopia-Somali (from 46.3 to 49%), Afar (from 52.5 to 54.5%), and Gambella (from 58.9 to 57.1%). Although Benishangul Gumuz has demonstrated a slight but significant decline in early childbearing (from 65.1 to 58.9%), the region carries by far the highest level of adolescent childbearing in the country.

### Determinants of early childbearing

The results of the multivariate Cox proportional hazard models that are presented in three separate models are shown in Table 3. The background factors included in the model were region, education, residence (urban/rural), and wealth (Model 1). Being the main proximate determinant of fertility, we added marital status to the background factors in Model 2. The final model (Model 3) further included contraceptive use in addition to the background factors and marital status, but the analysis was restricted to only married adolescents. The three models were fitted to the most recent cohort (i.e., 2000–2005).

**Table 3:** Adjusted hazard ratios (HRs) and *P*-values from multivariate Cox proportional hazard models showing the risk of early childbearing according to selected background and proximate factors in the 2000–2005 cohort in Ethiopia

	Cumulative probability (%) of early childbearing 2000–2005 (all women)	Model 1^a^ (all women)		Model 2^a^ (all women)		Model 3^a^ (women married before age 20 years)	
		Adjusted HR	*p*-value	Adjusted HR	*p*-value	Adjusted HR	*p*-value
Region							
Addis Ababa (ref)	9.6	1.0		1.0		1.0	
Tigray	35.9	3.5	0.000	2.08	0.000	1.48	0.008
Afar	54.5	3.06	0.000	1.64	0.004	1.19	0.282
Amhara	35.9	2.68	0.000	1.41	0.025	0.96	0.814
Oromia	40.4	3.38	0.000	1.87	0.000	1.41	0.014
Ethiopia-Somali	49.0	2.44	0.000	1.75	0.001	1.41	0.038
Benishangul-Gumuz	58.9	4.93	0.000	2.23	0.000	1.56	0.014
SNNP	31.9	2.7	0.000	1.95	0.000	1.49	0.006
Gambela	57.1	6.27	0.000	2.44	0.000	1.79	0.010
Harari	40.4	3.64	0.000	1.89	0.000	1.29	0.082
Dire Dawa	26.6	2.72	0.010	1.9	0.021	1.49	0.113
Residence							
Urban (ref)	18.8	1.0		1.0		1.0	
Rural	45.4	1.13	0.401	0.92	0.442	0.98	0.827
Education							
No education (ref)	56.2	1.0		1.0		1.0	
Primary	36.0	0.5	0.000	0.77	0.000	0.78	0.000
Secondary or higher	11.6	0.18	0.000	0.48	0.000	0.58	0.000
Wealth							
Poorest (ref)	49.6	1.0		1.0		1.0	
Poor	49.8	0.95	0.496	0.95	0.446	0.94	0.311
Medium	46.7	1.0	0.974	0.98	0.770	0.95	0.526
Rich	34.9	0.74	0.005	0.92	0.289	0.91	0.251
Richest	18.3	0.72	0.026	0.85	0.124	0.83	0.074
Marital status							
Married before age of 18 years (ref)	80.3			1.0		1.0	
Married at age of 18 years	46.1			0.4	0.000	0.39	0.000
Married at age of 19 years	16.2			0.22	0.000	0.22	0.000
Did not marry before age of 20 years	4.8			0.05	0.000		
Contraceptive used before age of 20 years							
No (ref)	26.1					1.0	
Yes	54.9					1.25	0.000

^a^Models adjusted for survey year and current age of the women; HR = adjusted hazard ratio; ref. = reference category

As shown in Model 1, the risk of early childbearing is significantly and independently associated with the region, woman’s education and household wealth. Compared with women in Addis Ababa, women in the other regions tended to have higher risks of early childbearing. The estimated likelihood of early childbearing ranged from 2.7–6.3 times higher in other regions compared with Addis Ababa. An inverse relationship can be noted between education and the risk of early childbearing. The risk of early childbearing among women with a primary education was 50% lower than those with no formal education. This decrease was even lower, by 82%, among women with a secondary or higher education. Household wealth was also associated with adolescent childbearing. The women from the richest households were 28% less likely than those from the poorest to have had early childbearing. Similarly, those from rich households had a 26% lower likelihood of early childbearing.

The multivariate results presented in Model 2 confirmed the apparent association between marriage (as measured by prevalence of marriage and age at marriage) and the likelihood of early childbearing. The risk of early childbearing decreased by 95% if the women remained unmarried until the age of 20 years compared with those women who married before the age of 18 years, which clearly signifies the very low level of out-of-wedlock childbearing in the country, as revealed by a 4.8% cumulative probability of early childbearing among unmarried adolescents vis-à-vis 80.3% for women who married before the age of 18 years. In addition, the findings from the same model suggest a decreased risk of early childbearing by 60 and 78%, respectively, for women who married at precisely 18 and 19 years of age compared with those who married before the age of 18 years.

The role of contraceptive use in the likelihood of early childbearing was assessed in a sub-sample of women who married during adolescence (Model 3). The model included use of contraceptives during the adolescent period along with age at marriage and background factors. It appears that married adolescents who practiced contraceptive use before the age of 20 years were 25% more likely than those who did not to have had early childbearing. This may well indicate a reverse causation in that married adolescents in our sample are more likely to adopt contraceptive use to space subsequent pregnancies than to completely avoid the first birth until the age of 20 years.

Notably, the inclusion of proximate factors, i.e., marital status and contraceptive use, in the models significantly modified the net effects of background factors on early childbearing. In Models 2 and 3, the net effects of the background factors, as measured by adjusted hazard ratios, were substantially reduced by the inclusion of marital status and contraceptive use. Of note, the net effect of household wealth on early childbearing waned after adjusting for proximate factors.

### Trends in early marriage

**Fig. 2:**
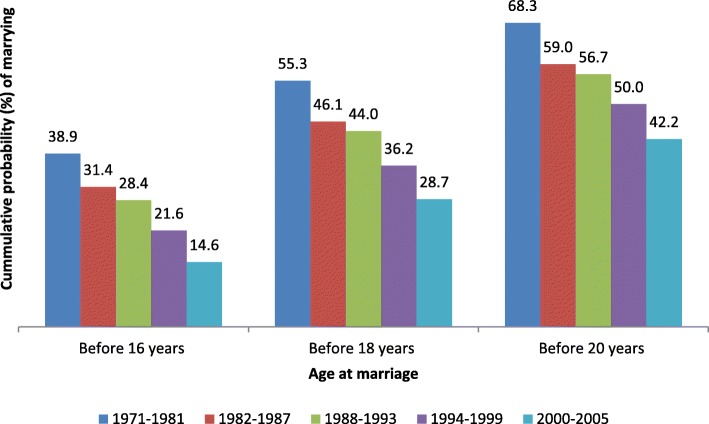
Cumulative probability of marrying before ages 16, 18 and 20 years by cohort in Ethiopia

As clearly demonstrated, the aforementioned analyses revealed that early marriage was perhaps the single most important proximate determinant of early childbearing in our analysis. Here, we present trends in early marriage before the ages of 16, 18 and 20 years, as shown in Fig. 2. The cumulative probability of marrying before the age of 20 years declined significantly (*p* < 0.000) from 68.3% in the 1971–1981 cohort to 42.2% in the 2000–2005 cohort. Likewise, the cumulative probability of marrying before the legal age of 18 years also dropped by nearly half from 55.3 to 28.7% (*p* < 0.000). The temporal decline was much more apparent for the incidence of marriages occurring before the age of 16 years, as it declined from 38.9 to 14.6% (*p* < 0.000) during the same period.

### Relationship between early marriage and early childbearing

**Fig. 3:**
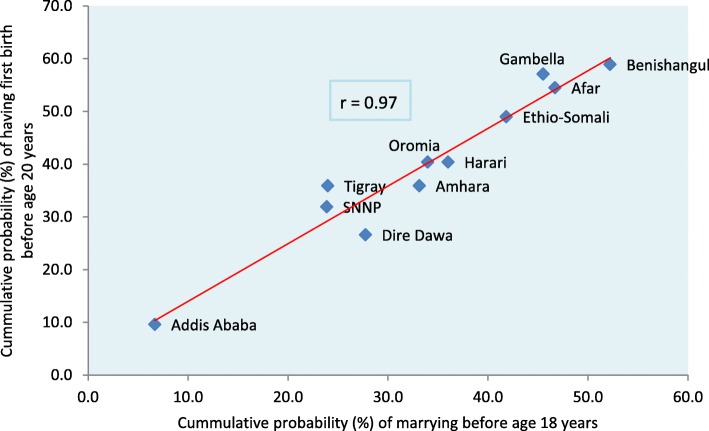
Cumulative probability of marrying before the age of 18 years vs. having first birth before the age of 20 years by region in the 2000–2005 cohort in Ethiopia

A scatter plot is presented in Fig. 3 to examine the correlation between early marriage (before the age of 18 years) and early childbearing (before the age of 20 years) using regional data from the most recent cohort. A very high and positive correlation (*r* = 0.97) can be noted between early marriage and early childbearing, in which regions with a high level of early marriage also exhibited a high level of early childbearing and vice versa. This finding further corroborated the aforementioned findings that early marriage is a major driver of early childbearing at national and regional levels. Indeed, the observed sub-national variation in early childbearing was largely explained by differences in the prevalence of early marriage across the regions.

## Discussion

This study was set out to examine trends, sub-national variations and determinants of early childbearing in Ethiopia. The analysis was based on nationally representative data from six rounds of surveys. The findings suggest that early childbearing, as measured by the cumulative probability of having a first birth before the age of 20 years, declined by approximately two-fifths from 57.6% for the 1971–1981 cohort to 35.3% for the 2000–2005 cohort. The decline was much more apparent in the recent period, especially for the cohort of women who reached puberty during 2000–2005, who in turn were in the age range of 20–26 years in 2016. This recent trend is plausible given that Ethiopia has made significant progress on many frontiers in the past two decades or so that are less conducive to adolescent motherhood. Important achievements that could affect early childbearing include the expansion of female education, community mobilization efforts and the promotion of collective community efficacy against harmful traditional practices, including marriage of girls and teenage pregnancy and childbearing, as well as enactment of the criminal code that prohibits marriage before the age of 18 years for girls [22]. In addition, the unprecedented improved population access to information and services on reproductive health and other health services through the health extension program and the stipulation of a number of policies and strategies on adolescents and youths should be emphasized. Undoubtedly, the synergies of these favorable social changes have contributed to the decline in early childbearing among Ethiopian adolescents in the recent period.

Previous studies in Ethiopia did not employ a similar cohort approach to track annual trends, as presented in this study, nor did they use the most recent surveys data in their analyses. This paper is thus a more distinctive and current reflection of past and present trends in adolescent childbearing in the country. Comparison of our findings with the few published studies from Ethiopia is not warranted due to methodological differences as well as the amount and nature of data used in our analyses. Nevertheless, there are indications from previous studies in Ethiopia of a decline in adolescent childbearing since the 1970s [15, 16].

Despite the recorded decline, however, adolescent motherhood is still fairly common in Ethiopia. Girls who reached puberty during the years from 2000 to 2005 had a 35% cumulative probability of becoming a teenage mother before their 20th birthday, and approximately one-fifth (19%) had already become teenage mothers before the age of 18 years. In comparison to most sub-Saharan Africa countries, however, Ethiopia can be considered among the countries with a relatively lower rate of adolescent childbearing. For example, UNFPA reported that among 30 sub-Saharan Africa countries in which 20% or more of women gave birth before 18 years of age, Ethiopia was among the five countries with relatively low rates of early childbearing, at less than 22%. In contrast, in most sub-Saharan countries, more than 25% of the female population has been reported to have given birth before 18 years of age; five of these countries have rates exceeding 40% [23]. Previous studies have also shown that in most regions of Sub-Saharan Africa, adolescent childbearing has declined at a moderate pace over the past few decades; however, only a few countries in the region have recorded faster declines in adolescent fertility [24, 25].

Marriage determines fertility levels and patterns in societies in which most childbearing occurs within marriage [26]. Our analyses confirmed that the prevalence of marriage during adolescence and variation in age of entry into marriage were by far the most important predictors of early childbearing in Ethiopia. The risk of early childbearing decreased by 95% if women remained unmarried until their 20th birthday vis-à-vis those who married before the age of 18 years. This result clearly underscores that adolescent pregnancy and birth in Ethiopia occur largely within the context of marriage and that out-of-wedlock childbearing can be considered uncommon among the adolescents in the country. Other studies have reported similar findings for the relationship between early marriage and early childbearing [27, 28]. Furthermore, we showed that in addition to the virtual absence of marriage during the adolescent period, the age of entry into marriage appeared to significantly alter the likelihood of early childbearing. Marrying at the legal age of 18 years can reduce the risk of early childbearing by 60% in comparison to marrying before the age of 18 years. A shift of the age at marriage to 19 years reduced the risk of early childbearing by 78%. These interesting findings are central to explaining the recorded trends in early childbearing in the past four decades in the country. Analysis of the cohort data on the prevalence and age pattern of marriage among adolescents divulged significant declining trends over the past four decades that are compatible to the recorded parallel decline in early childbearing in the country. In the past four decades, the cumulative probability of early marriage (i.e., before the legal age of 18 years) declined by approximately half, from 55.3 to 28.7%.

In 2005, Ethiopia adopted a criminal law that enforces the minimum legal age of marriage for women to 18 years [29]. The code also allows the termination of pregnancy under special conditions, including when the pregnancy is a result of rape, when it endangers the life of the mother or the child or if the pregnant woman is physically or mentally unfit to raise a child, including unwanted pregnancy in teenagers. The Ethiopian Women Lawyers Association, in collaboration with the Ministry of Justice and local and international NGOs and community-based organizations, campaigns for communities, local governments and religious institutions to observe the legal age of 18 for marriage. They also provide legal literacy training on the issue of early marriage. Communities have been reached through the educational efforts of health workers, women’s associations, teachers and religious leaders. Legislative bodies and other influential groups in communities have been sensitized to enact and implement laws that protect women and girls from early marriage and other harmful traditional practices [22]. Nevertheless, a common criticism of the marriage law has been the lack of strong and wider implementation and enforcement, especially in rural parts of the country where early marriage and childbearing are common and where more than three-quarters of the population resides. One study that focused on early marriage concluded that knowledge of and respect for the marriage law is limited among many rural communities in Ethiopia [22]. Despite the law, the analysis presented in this paper also signals a fairly high incidence of early marriage in the recent cohort, with the cumulative probability of marrying before the age of 18 years estimated at 28.7% from 2000 to 2005. Based on this study, it is also unknown if and how the marriage law has impacted the recorded decline in early childbearing in recent years. Most importantly, our analyses underscore that a mere shift of the legal age at marriage to 18 years could significantly reduce the risk of early childbearing by 60%, which reinforces the significant relevance of the wider application of the marriage law for the health and wellbeing of adolescents in the country. Our study corroborates a previous study conducted in 12 sub-Saharan African countries that showed a lower prevalence of teenage childbearing in countries with a consistent minimum marriage age law of 18 years compared with countries without consistent laws [30].

In agreement with previous studies in Ethiopia [15–17] and elsewhere [25, 27, 28], we observed a lower risk of early childbearing with an increase in women’s education. We found a 50 and 82% lower risk of early childbearing among women with elementary and secondary or higher education, respectively, compared with women with no education. Other studies of sub-Saharan Africa reported similar results. For example, Gupta et al. (2003) concluded that the education of girls from about the secondary level onwards is a key instrument for raising the age at first birth in sub-Saharan Africa but suggest that increases in schooling at lower levels alone bear only marginal effects on prospects for fertility decline among adolescents [31]. The relationship between education and adolescent fertility could be mediated by several factors. Girls with a higher education level have a lower probability of having a child before their 20th birthday mainly because of an older age at marriage. Education presents girls with more incentive to avoid early childbearing because they can achieve greater exposure and access to sexual reproductive information and receptivity to birth control [32]. Additionally, education often empowers girls with improved skills in family communication about sexual and reproductive health and other social issues, as well as their level of control within the family [33]. In recent decades, the participation of Ethiopian adolescents in primary or secondary education has improved remarkably from less than 40% in 2000 [34] to 90% in 2016 [21]. It is therefore plausible that the increase in women’s education in the past two decades in Ethiopia has inhibited adolescent fertility mainly through delayed marriage but also, to some extent, through improved female empowerment to make decisions concerning their sexual and reproductive health.

Contraceptive use among married adolescents in Ethiopia has increased substantially over the past few decades, from a low of 2% in 1990 [35] to 34% in 2016 [21]. However, contraceptive use did not emerge as a significant inhibitory factor for delaying first birth until the age of 20 years among married adolescents in our analysis. In contrast to expectations, it appears that married adolescents who practiced contraceptive use before the age of 20 years were 25% more likely than those who did not to have had early childbearing. This finding should be interpreted with caution, as it represents a reverse causation and suggests that married adolescents are more likely to use contraceptives in order to space subsequent pregnancies than to delay first birth until the age of 20 years. The observed lower adoption of contraception before first birth by married adolescents is not unique to this paper, and a recent study examining the reproductive behaviors of adolescents in rural Ethiopia observed lower contraceptive use before first birth than after first birth [36]. This finding is further corroborated by studies conducted in sub-Saharan Africa and South Asia, suggesting that married adolescents are typically under pressure from their husbands, their in-laws and their communities to begin childbearing immediately after marriage [37, 38]. For married teens, it is often important to prove their fertility as soon as they are married [37], and this reality has been reflected in DHS surveys that found married teens are not only less likely to use contraception to delay first birth but also have a reduced unmet need for contraception [38]. In most of these societies, infertility is deeply feared by most married women [39] because it can result in divorce and abuse by their husbands.

Stark sub-national variations in adolescent motherhood have persisted over the years, and the most recent cohort of women revealed a cumulative probability of early childbearing that ranged from a low of 9.6% in Addis Ababa to 58.9% in Benishangul Gumuz. In six of the 11 regions, early childbearing probabilities exceeded 40%, and three of them exceeded 50%. Additionally, the decline in early childbearing recorded at the national level was not shared equally across the regions. While five regions registered a significant decline in early childbearing over the years, no such trend was recorded in the other regions. There are also regions such as Ethiopia-Somali, Afar and Gambella that have demonstrated a staggeringly stable or reverse trend in early childbearing since the early 1970s. The very high correlation we observed between early marriage and early childbearing (*r* = 0.97) is relevant for our understanding of the source of variations in early childbearing across regions. Our analysis confirmed that regions with a high rate of early marriage tended to have a correspondingly high rate of early childbearing, and vice versa. Akin to early childbearing, early marriage rates also varied greatly across the regions, ranging from 6.7% in Addis Ababa to 52.2% in Benishangul Gumuz. Early marriage disparities across regions could be a reflection of differences in the socio-cultural and economic situations of the regions, such as differences in religion, traditional norms and the lack of inter-regional uniformity in the implementation and enforcement of the marriage law, among other factors. Further studies are warranted to unravel the sources of variations in early marriage and childbearing across the regions.

This paper has strengths that merit discussion. First, this study was based on six nationally representative surveys that were collected over the past 16 years and that can easily be compared over time and provide a large data set to allow robust statistical analyses. The three rounds of the DHS and three rounds of the PMA surveys are similar in the following aspects: the surveys used a similar sample design; sampling was performed by the same agency – the Ethiopia Statistical Agency; and similar questions were used in all rounds of the surveys for all variables studied in this paper. Second, we employed a robust approach to explore annual trends in adolescent childbearing and marriage experiences that, to our knowledge, have not been previously used in the Ethiopian context. This method permits a better understanding of how these important events have been changing over time. Third, we estimated cumulative probabilities instead of percentages to overcome the problem of censoring of adolescents who have not yet experienced events such as childbirth or marriage. Indeed, the percentage of adolescents aged 15 to 19 years who have had a live birth is affected by censoring because those girls without a live birth still face the risk of pregnancy before they reach age 20 years.

Despite the aforementioned strengths, this paper has limitations that should be mentioned. First, recall bias on the age and timing of first birth, first marriage and first contraceptive use may have been present in the data and cannot be discounted. The recall biases for these events may have been higher among older women because these events were more likely to have occurred many years ago. However, we do not feel that these biases significantly affected the present findings and conclusions of this study because the directions of the potential biases are unknown and are more likely to be random. Second, due to the obvious cross-sectional nature of the DHS and PMA surveys, causal relationships between events could not be assumed, and it was impossible to establish a temporal sequencing of events.

## Conclusion

This study provides compelling evidence for a substantial reduction in early childbearing over the past four decades in Ethiopia, which was driven largely by a parallel and significant reduction in the prevalence of early marriage during the same period. However, a fairly large portion of Ethiopian adolescents are still suffering from early childbearing; and the situation appears to be more dismal in some regions compared with others.

Childbearing occurs predominantly within the milieu of marriage in Ethiopia, and as a result, the prevalence of marriage and the age of entry into marriage during the period of adolescence are by far the most important determinants of early childbearing in the country. Our analysis underscores that postponing marriage to the legal age of 18 years and beyond can substantially reduce the risk of early childbearing. Thus, a further decline in early childbearing could be achieved by enforcing the Ethiopian law on the minimum marriage age, which entails a continued education of the general public and communities about the law, involving community leaders, legislative bodies and local governments to seriously observe its implementation. Our analysis also confirms the potential of female education, especially secondary or higher levels of education, to curb the incidence of early childbearing among Ethiopian adolescents. Thus, further expansion of female education from the secondary level onwards and a reduced school dropout rate of girls will have a greater role in future prospects of fertility decline among adolescents in the country.

Married adolescents often find it difficult, if not impossible, to delay the first birth until the age of 20 years due to the social pressure supporting childbirth shortly after marriage. Despite the challenges, married adolescents and their partners should be provided with the necessary information and services to help them delay early childbearing. Family planning programmers must devise ways to reach out to newly married adolescents with the necessary information and services before they initiate childbearing. Priority interventions to address early childbearing in the country should also focus on dispelling community norms and attitudes that encourage early childbearing among married adolescents.

Finally, the pervasive sub-national variations in adolescent fertility, which is largely due to differences in the incidence of early marriage, calls for region-specific interventions to address the socio-economic and cultural factors that are pertinent to the regions.

## Abbreviations

CSA: Central Satirical Agency
DHS: Demographic and Health Survey
EDHS: Ethiopia Demographic and Health Survey
EPMA: Ethiopia Performance Monitoring and Accountability
HR: Hazard Ratio
NGO: Non-Governmental Organization
PMA: Performance Monitoring and Accountability
USAID: United States Agency for International Development

## References

[1] The Lancet Commissions. Our future: a lancet commission on adolescent health and wellbeing. Lancet. 2016;387:2355-6. 10.1016/S0140-6736(16)00579-1 PMC5832967 27174304

[2] World Health Organization: Adolescent Pregnancy Fact Sheet; 2012. http://www.who.int/en/news-room/fact-sheets/detail/adolescent-pregnancy.

[3] United Nations Population Fund (2013). Motherhood in childhood: facing the challenge of adolescent pregnancy: New York, UNFPA.

[4] Gupta N, Leite CI (1999). Adolescents fertility behaviour: trends and determinants in Northern Brazil. Int Fam Plan Perspect.

[5] Save the Children (2012). Charting the future: Empowering girls to prevent early pregnancy: UK, Save the children.

[6] Patton G, Coffey C, Sawyer S, Viner R, Haller D, Bose K, Vos T, Ferguson J, Mathers C (2009). Global patterns of mortality in young people: a systematic analysis of population health data. Lancet.

[7] World Bank. Development and the next generation, world development report. In: International Bank for Reconstruction and Development. Washington DC: World Bank; 2007.

[8] Kennedy E, Gray N, Azzopardi P, Creati M (2011). Adolescent fertility and family planning in East Asia and the Pacific: a review of DHS reports. Reprod Health.

[9] United Nation Population Fund. The state of world population 2016. New York: UNFPA; 2016.

[10] Central Statistical Agency [Ethiopia] and ICF International. Ethiopia Demographic and Health Survey 2011. Addis Ababa, Ethiopia and Calverton, Maryland: ORC Macro; 2012.

[11] United Nations (2015). World Fertility pattern 2015 - data booklet.

[12] Federal Ministry of Health [Ethiopia]. Health Sector Transformation Plan:2015-2020. Addis Ababa: FMOH; 2015.

[13] Federal Ministry of Health [Ethiopia]. National Reprod Health Strategy 2016–2020. Addis Ababa: FMOH; 2016.

[14] Sedgh G, Finer L, Bankole A, Eilers M, Singh S (2015). Adolescent pregnancy, birth, and abortion rates across countries: levels and recent trends. J Adolesc Health.

[15] Gurmu E, Dejene T (2012). Trends and differentials of adolescent motherhood in Ethiopia: evidences from 2005 demographic and health survey. Afr J Reprod Health.

[16] Gurmu E, Etana D (2014). Age at first marriage and first birth interval in Ethiopia: analysis of the roles of social and demographic factors. Afr Popul Stud.

[17] Mekonnen W. Differentials of Early Teenage Pregnancy in Ethiopia: 2000 and 2005. Maryland: ICF international Calverton. p. 2013.

[18] Lindstrom D, Kiros G, Hogan D (2009). Transition into first intercourse, marriage, and Childbearing among Ethiopian Women. Genus.

[19] Reda A, Lindstrom DP (2014). Recent trends in the timing of first sex and marriage among young women in Ethiopia. Afr Popul Stud.

[20] The Demographic and Health Survey Program DHS overview. http://dhsprogram.com/What-We-Do/Survey-Types/DHS.cfm

[21] Performance Monitoring and Accountability 2020 Project, School of Public Health Addis Ababa University: Detailed Indicator Report: Ethiopia 2016. Baltimore: PMA; 2016.

[22] Alemu B. Early marriage in Ethiopia: causes and health consequences. Kampala: International Center for Research on Women; 2007.

[23] United Nation Population Fund (2013). Adolescent pregnancy: a review of the evidence.

[24] United States Bureau of the Census (1996). Trends in adolescent fertility and contraceptive use in the Developing World.

[25] Mahy M, Neeru G (2002). Trends and Differentials in Adolescent Reproductive Behavior in Sub-Saharan Africa DHS Analytical Studies.

[26] Fargues P, Makhlouf Obermeyer C (1995). Changing hierarchies of gender and generation in the Arab world. Family, gender and population in the Middle East.

[27] Zamilum M, Salim M (2013). Patterns and determinants of age at first birth in Bangladesh. Turkish Journal of Population Studies.

[28] Nahar Q, Min H (2008). Trends and Determinants of Adolescent Childbearing in Bangladesh.

[29] Alemu B: How to end child marriage: action strategies for prevention and protection. International Center for Research on Women; 2007.

[30] Maswikwa B, Richter L, Kaufman J, Nandi A (2015). Minimum marriage age Laws and the prevalence of child marriage and adolescent birth: evidence from sub-Saharan Africa. Int Perspect Sex Reprod Health.

[31] Gupta N, Mahy M (2003). Adolescent childbearing in sub-Saharan Africa: can increased schooling alone raise ages at first birth?. Demogr Res.

[32] Manlove J, Terry L, Gitelson A, Papillo Y, Russell S (2000). Explaining demographic trends in teenage fertility, 1980–1995. Fam Plan Perspect.

[33] di Cesare M, Rodríguez VJ (2006). Micro analysis of adolescent fertility determinants: the case of Brazil and Colombia. Papeles de Población.

[34] Central Statistical Authority and ICF International: Ethiopia demographic and health survey 2000*.* Addis Ababa, Ethiopia and Calverton, Maryland, USA: Central Statistical Agency; 2000.

[35] Central Statistical Agency. The 1990 Family and fertility survey. Addis Ababa: Central Statistical Agency; 1991.

[36] Null C, Smith K, Sridharan S, Mekonnen Y, Wolde E (2017). Promoting a community-based response to the reproductive health and livelihood needs of married adolescent girls and young women in Ethiopia: Evaluation findings and leanings.

[37] Wood K, Jewkes R (2006). Blood blockages and scolding nurses: barriers to adolescent contraceptive use in South Africa. Reprod Health Matters.

[38] Khan S, Mishra V (2008). Youth reproductive and Sex Health, DHS Comparative Reports No. 19.

[39] Lloyd C, National Research Council and Institute of Medicine (2005). Growing Up Global: The changing transitions to adulthood in developing countries.

